# A Combined Marker of Inflammation in Individuals with Mania

**DOI:** 10.1371/journal.pone.0073520

**Published:** 2013-09-03

**Authors:** Faith Dickerson, Cassie Stallings, Andrea Origoni, Crystal Vaughan, Emily Katsafanas, Sunil Khushalani, Robert Yolken

**Affiliations:** 1 Stanley Research Program, Sheppard Pratt Health System, Baltimore, Maryland, United States of America; 2 Department of Pediatrics, Johns Hopkins School of Medicine, Baltimore, Maryland, United States of America; University of Adelaide, Australia

## Abstract

**Background:**

Markers of immune activation have been associated with mania but have not been examined in combination. We studied the association between mania and an inflammation score based on four immune markers.

**Methods:**

A total of 57 individuals with mania were assessed at up to three time points: the day of hospital admission, evaluation several days later, and six-month follow-up. Also assessed were 207 non-psychiatric controls and 330 individuals with recent onset psychosis, multi-episode schizophrenia, or bipolar disorder depression. A combined inflammation score was calculated by factor analysis of the levels of class-specific antibodies to the NR peptide of the NMDA receptor; gliadin; Mason-Pfizer monkey virus protein 24; and *Toxoplasma gondii*. Inflammation scores among groups were compared by multivariate analyses. The inflammation score of the mania group at evaluation was studied as a predictor of re-hospitalization in the follow-up period.

**Results:**

The combined inflammation score of the mania group at hospital admission and at evaluation differed significantly from that of the non-psychiatric controls (t = 3.95, 4.10, p<.001). The inflammation score was significantly decreased at six month follow-up (F = 5.85, p = 0.004). There were not any significant differences in the inflammation scores of any of the other psychiatric groups and that of the controls. Within the mania group, an elevated inflammation score at evaluation predicted re-hospitalization (Hazard ratio = 7.12, p = .005).

**Conclusions:**

Hospitalization for mania is associated with immune activation. The level of this activation is predictive of subsequent re-hospitalization. Interventions for the modulation of inflammation should be evaluated for the therapy of individuals with mania.

## Introduction

Mania is an abnormal mood state and the defining characteristic of bipolar disorder. The etiology of mania is largely unknown. Although genetic factors play a major role in the etiology of bipolar disorder, most have relatively low odds ratios [1], [2]. Immunological abnormalities have been identified and may contribute to the pathophysiology of mania as well as to bipolar disorder more broadly [3]–[7]. Such factors may help explain the marked fluctuations in mood which are the hallmark of the disorder [8].

A number of infectious and inflammatory markers have been associated with episode of mania. In a previous study, we found that individuals with mania had increased levels of antibodies to the NR2 peptide of the NMDA receptor at time of hospital admission and also several days later during the hospital stay, but not at a six month follow up, compared to a non-psychiatric control group and also compared to several psychiatric comparison groups [9]. In these same individuals with mania we also found elevated levels of IgG antibodies to gliadin, which is derived from the wheat protein gluten, but not to other markers of celiac disease [10]. Again the antibodies were elevated during hospital admission but not at a six month follow-up. Additionally we found elevated levels of IgM antibodies to *Toxoplasma gondii* in individuals with mania. *Toxoplasma gondii i*s a protozoan of the family apicomplexa; IgG antibodies to *Toxoplasma gondii* have been associated with schizophrenia in previous studies [11]. The finding of IgM class antibodies suggests a recent exposure or reactivation of the organism. In a study of individuals with a recent onset of psychosis, we found elevated levels of antibodies to an animal retrovirus, Mason-Pfizer Monkey Virus, compared to non-psychiatric controls [12]. Such elevated antibody levels are likely to have been derived from the activation of homologous endogenous retroviruses and to be a reflection of immune activation. Evidence of activation of endogenous retroviruses has also been found in other individuals with bipolar disorder [13].

Previous studies have focused on the role of individual markers of immune activation. In this study we sought to examine the role of multiple immunological determinants by use of a combined inflammation factor score based on the levels of four immune markers shown to have individual associations with bipolar disorder. It is the combination of markers that is likely to provide the strongest clinical associations. We compared the combined inflammation scores in the mania group with those in a non-psychiatric control group and in other psychiatric comparison groups. We also examined the combined inflammation score as a predictor of re-hospitalization in the individuals with mania.

## Methods

### Participant Recruitment and Characterization

The study sample consisted of 594 unique individuals in five groups: 57 hospitalized for symptoms of mania; 28 hospitalized with bipolar disorder depression; 68 with a recent onset of psychosis; 234 with multi-episode schizophrenia; and 207 controls without a history of psychiatric disorder.

Individuals with mania were recruited from inpatient and day hospital programs at a large psychiatric hospital in the Baltimore, MD region. Inclusion criteria for the mania participants were: current admission to an inpatient or day hospital program for symptoms of mania or hypomania and potentially available for in-person follow-up six months later. Participants with mania could have an admission diagnosis of any one of the following: Bipolar I disorder, single manic episode; Bipolar I disorder, most recent episode manic; Bipolar I disorder, most recent episode mixed; Bipolar II disorder, most recent episode hypomanic; or Schizoaffective disorder, bipolar type (manic, hypomanic, or mixed state). Individuals with bipolar disorder depression were recruited from persons admitted to an inpatient or day hospital unit at the same psychiatric hospital for symptoms of depression with an admission diagnosis of bipolar disorder. Individuals with a recent onset of psychosis were recruited from inpatient and day hospital programs also at the same psychiatric hospital as well as from a local program for persons with a first episode of psychosis. Inclusion criteria for the recent onset of psychosis participants were: the onset of psychotic symptoms for the first time within the past 24 months (defined as the presence of a positive psychotic symptom of at least moderate severity that lasted through the day for several days or occurred several times a week and could not have been limited to a few brief moments) and aged 18–45. Individuals with multi-episode schizophrenia, not of recent onset, were recruited from local psychiatric treatment centers as well as from inpatient and day hospital programs at the psychiatric hospital where the other patient groups were recruited. Inclusion criterion for this group was a diagnosis of Schizophrenia or Schizoaffective disorder. Individuals without a history of psychiatric disorder were recruited from posted announcements at local health care facilities and universities in the same geographic area as the settings where the psychiatric participants were recruited. These control individuals were enrolled after they were screened to rule out the presence of a current or past psychiatric disorder with the Structured Clinical Interview for DSM-IV Axis I Disorders- Non-patient Edition [14]. Participants in all groups met the following additional criteria: age 18–65 (except the recent onset participants who were aged 18–45, and the control participants who were aged 20–60); proficient in English; absence of any history of intravenous substance abuse; absence of mental retardation; absence of HIV infection; absence of serious medical disorder that would affect cognitive functioning; absence of a primary diagnosis of alcohol or substance use disorder. The diagnosis of each psychiatric participant was established by consensus of the research team based on the SCID for DSM-IV Axis 1 Disorders – Patient Edition [15] and available medical records. For the purposes of this study, participants were only included in one psychiatric group with the order of selection: mania, bipolar disorder depression, recent onset psychosis, multi-episode schizophrenia.

The studies were approved by the Institutional Review Boards of the Sheppard Pratt Health System and the Johns Hopkins Medical Institutions following established guidelines. All participants provided written informed consent after the study procedures were explained.

Participants were asked about their educational level and other demographic variables including age at illness onset and maternal education as a proxy for pre-morbid socioeconomic status. All participants were individually administered a brief cognitive battery, the Repeatable Battery for the Assessment of Neuropsychological Status, Form A (RBANS) [16]. All of the psychiatric participants were also interviewed and rated on the Positive and Negative Syndrome Scale (PANSS) [17]. Additionally, the mania participants and the bipolar disorder depression participants were assessed on mood rating scales: the Young Mania Rating Scale (YMRS) [18] and the Hamilton Rating Scale for Depression (Ham-D) [19]. For the mania patients, this clinical evaluation took place at the time of the baseline visit during the hospital stay and at the six month follow-up. For other participants, data were obtained at the one study visit. For all of the psychiatric participants, psychiatric medication data were recorded from participant self-report and clinical charts and it was noted whether each patient was receiving each of the following types of medication at the time of the study visit: lithium, anti-convulsant mood stabilizer, second generation anti-psychotic, and/or anti-depressant medications.

### Laboratory Evaluations

A blood sample was obtained at three time points for most mania participants: at the time of hospital admission (from archived samples that were collected as part of the admission medical work-up); at the time of consent to the study and baseline evaluation during the hospital stay; and at the time of the six month follow-up. A blood sample was obtained at the study visit for the other groups: recent onset of psychosis, multi-episode schizophrenia, bipolar disorder depression and non-psychiatric controls.

Four antibody markers were used to calculate the combined inflammation score: 1) IgG class antibodies to the NR2 peptide fragment of the NMDA receptor as previously described (9); 2) IgG class antibodies to gliadin as previously described [10]; 3) IgG class antibodies to the Mason-Pfizer monkey virus gag protein as previously described [12]; 4) IgM class antibodies to *Toxoplasma gondii.* [20] These antibodies were measured by solid phase immunoassay techniques.

### Data Analyses

A principal components factor analysis without rotation was used to calculate a combined inflammation factor score based on the four immune markers in all of the samples [21]. Linear regression models with robust standard errors were used to compare the level of the combined inflammation score in each of the 6 study groups (mania at three time points, recent onset psychosis, multi-episode schizophrenia, bipolar disorder depression) from that of the reference group, the non-psychiatric controls. Logistic regression models were used to calculate the odds ratios associated with an increased level of the combined inflammation score in each of the psychiatric study groups; an increased level of antibodies was defined as a combined inflammation factor score> = 75^th^ percentile of that in the controls and > = 90^th^ percentile of that in the controls. All models were adjusted for age, gender, race, and level of maternal education.

Within the mania group one-way analysis of variance was used to examine the association between the combined inflammation score and clinical variables and types of medication received. Repeated measures analysis of variance was used to determine the change in the level of the combined score at the three sampled time points.

Also within the mania group Cox proportional hazards analysis was used to examine the association between an increased level of antibodies (> = 90^th^ percentile of the combined score of the controls) and re-hospitalization in the follow-up period. Rehospitalization was defined as admission to the hospital following at least a two week interval since the previous discharge. These analyses included the covariates age, gender, race, maternal education, and PANSS total symptom score at the time of the hospital evaluation.

All statistical analyses were performed with STATA version 11, College Station, Texas.

## Results

The demographic and clinical characteristics of the five study populations are presented in **Table 1**. The medications that psychiatric participants were receiving at the time of the study evaluation are shown in **Table 2**.

**Table 1:** Demographic and clinical characteristics of study populations (N = 594).

	Mania (n = 57)^1^	Recent onsetpsychosis (n = 68)	Multi-episode schizophrenia (n = 234)	Bipolar disorder depressed (n = 28)	Non-psychiatric controls (n = 207)
Age, years	35.1 (12.7)	23.5 (5.6)	41.5 (11.7)	33.0 (13.3)	32.3 (11.4)
Gender male	16 (28%)	44 (64%)	137 (59%)	6 (21%)	80 (39%)
Race white	37 (65%)	31 (46%)	118 (50%)	22 (79%)	117 (57%)
Years education^2^	14.2 (2.2)	13.0 (2.2)	12.1 (2.5)	12.7 (1.9)	15.5 (2.5)
Years maternal education^3^	13.7 (2.8)	13.9 (2.8)	12.4 (2.7)	13.2 (3.6)	13.7 (2.6)
RBANS^4^ total	74.8 (13.0)	70.0 (14.1)	63.8 (11.7)	77.6 (13.5)	86.2 (12.1)
PANSS^5^ total score	75.8 (11.6)	77.9 (14.7)	74.0 (13.8)	69.4 (9.5)	–
YMRS^6^ total score	18.6 (8.2)	–	–	6.7 (6.1)	–
Ham-D^67^ total score	19.6 (8.8)	–	–	28.8 (7.4)	–

1 Participants with mania were assessed at time of evaluation during the hospital stay.

2 Multi-episode schizophrenia, n = 233.

3 Multi-episode schizophrenia, n = 228; Non-psychiatric controls, n = 205.

4 RBANS = Repeatable Battery for the Assessment of Neuropsychological Status.

5 PANSS = Positive and Negative Syndrome Scale.

6 YMRS = Young Mania Rating Scale.

7 Ham-D = Hamilton Rating Scale for Depression.

**Table 2:** Psychiatric medications received by study participants.

	Mania (n = 57)^1^	Recent onsetpsychosis (n = 68)	Multi-episodeschizophrenia (n = 234)	Bipolar disorderdepression (n = 28)
Lithium	21 (37%)	17 (25%)	32 (14%)	11 (39%)
Second generation antipsychotic	42 (74%)	61 (90%)	189 (81%)	19 (68%)
Anti-depressant	11 (19%)	24 (35%)	89 (38%)	21 (75%)
Anticonvulsant including lithium	55 (96%)	22 (32%)	107 (46%)	22 (79%)

1 Medications were recorded for the mania group at time of evaluation during the hospital stay. For all other groups, the medications are those received at the time of the one study visit.

All 57 mania participants were seen at the baseline evaluation visit. The diagnoses of the mania participants were: bipolar I disorder type, most recent episode manic, n = 28 (49%); most recent episode mixed, n = 21 (37%); bipolar I disorder, single manic episode, n = 2 (4%); bipolar II disorder, n = 1 (2%); schizoaffective disorder, n = 5 (9%). A total of 44 of 57 (77%) also had a hospital admission blood sample which was drawn on average 4.0 (s.d. 2.3) days before the evaluation sample. A total of 38 of 57 (67%) mania participants were evaluated at a six month follow-up visit and had a blood sample drawn at that time. The mean time between enrollment and follow up was 191.0 days (s.d. 12.9 days). The mean YMRS (Young) score at the baseline evaluation was 18.6 (s.d. 8.2) and at the six month follow-up was 7.4 (s.d. 6.8); it is of note that the baseline evaluation was performed several days after hospital admission, so may not reflect the participant’s acuity at the time of admission. A total of 29 (51%) of the mania participants had samples from all 3 time points.

The four markers and the combined inflammation score were available for all of the study participants. The results of the linear regression comparing the level of the combined inflammation score in each of the psychiatric study groups to that of the reference group, the non-psychiatric controls, indicates a significant difference from the reference group only for the mania group at time of hospital admission (t = 3.95, p<.001) and at time of evaluation during the hospital stay (t = 4.10, p<.001) and not for the mania group at the six month follow-up or for any of the other psychiatric groups (all p>.05).

**Figure 1:**
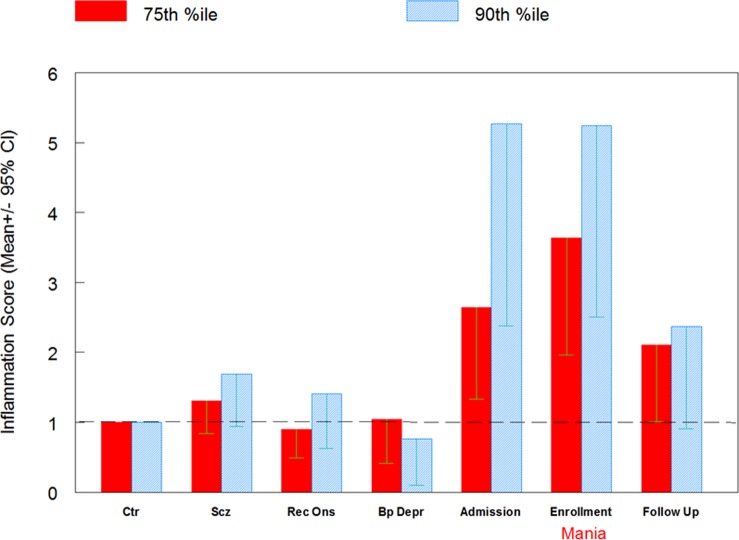
Odds ratios of elevated levels of the combined inflammation score in the mania group at three time points and in psychiatric comparison groups. Ctr = non-psychiatric controls (n = 207), Scz = multi-episode schizophrenia (n = 234), RecOns = recent onset of psychosis (n = 68), BpDepr = Bipolar disorder depression (n = 28), Mania at admission (n = 44, Mania at enrollment (n = 57), Mania at follow up (n = 38). 75^th^ %tile represents > = the 75^th^ percentile of the controls 90^th^ % tile represents > = the 90^th^ percentile of the controls.

The results of the logistic regression analysis used to calculate the odds ratios associated with an increased level of the combined inflammation score are depicted in **Figure 1**. Using a cutoff of > = 75^th^ percentile of that of the controls there was a significantly increased odds for the mania group at time of hospital admission (OR = 2.79, 95% CI 1.33, 5.23, p = .005) and at time of evaluation during the hospital stay (OR 3.65, 95% CI 1.96, 6.79, p<.001) and also a marginally significant increased odds at time of the six month follow-up (OR 2.11, 95% CI 1.01, 4.40, p = .046). Using a cutoff of > = 90^th^ percentile of that of the controls there was also significantly increased odds for the mania group at time of hospital admission (OR 5.27, 95% CI 2.39, 11.63, p<.001) and at time of evaluation during the hospital stay (OR 5.25, 95% CI 2.52, 10.95, p<.001) but not significant odds for the mania group at the six month follow-up or for any of the other psychiatric groups. Levels of the combined inflammation score were significantly decreased in the mania group at follow-up as measured by repeated measures analysis of variance (F = 5.85 p = 0.004).

Within the mania group there was not a significant association at time of the evaluation visit between the combined inflammation score and any of the clinical variables (PANSS total, YMRS score, Ham-D score, RBANS score) or type of medication received (second generation antipsychotic, anti-convulsant mood stabilizer, lithium, antidepressant) (all p>.05).

**Figure 2:**
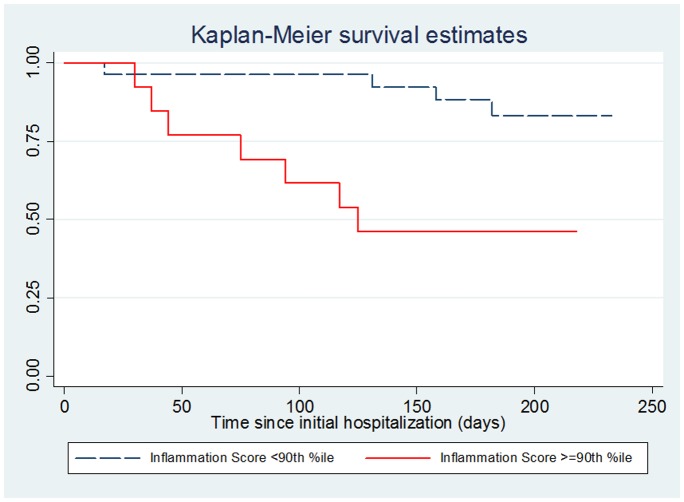
Combined inflammation score and re-hospitalization in individuals with mania. A total of 11 of 38 patients assessed at follow-up were re-hospitalized during the six month follow-up period. An elevated inflammation score was defined as> = the 90^th^ percentile of the level of the combined inflammation score in the non-psychiatric control group**.**

A total of 11 participants were re-hospitalized in the six month follow-up period (29% of the 38 individuals who were evaluated at the 6 month follow-up). Results of a Cox Proportional Hazards analysis looking at the association between an increased level of the combined inflammation score (> = 90^th^ percentile of the level of the controls) at the time of hospital evaluation and re-hospitalization during the six month follow-up period shows a significant association (Hazard ratio = 7.12, p = <.005) as shown in **Figure 2**.

## Discussion

We found that individuals with mania had increased levels of a combined inflammation score, based on four antibody markers, at time of hospital admission and at time of evaluation during the hospital stay compared to a control population. The individuals with mania at hospital admission and at evaluation during the hospital stay also had significantly increased odds of having a combined inflammation score that was greater than the 75^th^ percentile and the 90^th^ percentile level of this score in the control group. It is of note that the levels in the mania group were found to be significantly decreased at time of the six month follow-up compared to during the hospital stay. This finding indicates that high levels of immune activation occur in many individuals with mania at the time of hospitalization and subsequently decline. We also found that an increased inflammation score predicted re-hospitalization in individuals with mania within a six month period of time. Our findings are consistent with the concept of increased inflammation during acute episodes of mania [3]–[5] and expand on our findings with the individual markers [9], [10].

The reasons for the elevated combined inflammation score in individuals with mania are not known with certainty. However, it is likely that this elevated score represents an increase in immune activation occurring in individuals with mania around the time of their hospitalization. This activation could represent an exposure to an exogenous infectious agent, a reactivation of an endogenous agent, or the progression of an autoimmune reaction. The fact that we did not find elevated levels of the combined inflammation score in any of our non-mania psychiatric comparison groups also suggests that the finding is specific to individuals in an acute manic state. The fact that the individuals with bipolar disorder depression were also hospitalized and were generally receiving the same classes of psychiatric medications as the individuals who were hospitalized for mania suggests that treatment in the hospital setting, per se, does not account for this result.

It is of note that we did not find an association between the combined inflammation score and the severity of symptoms within the mania group during the hospital stay. Because we did not measure symptom scores at hospital admission, presumably the time of highest symptom acuity, we do not know if differences would have been found at this time. Future studies would also benefit from investigating the association between the duration of the mania episode and the level of inflammation markers.

Strengths of our study include the relatively large total sample size, the number of the comparison groups, and that all of the participants were drawn from the same geographic area and, in the case of the psychiatric participants, the same psychiatric healthcare system. In addition, the fact that our mania group was sampled longitudinally enables us to conclude with some confidence that the elevations in combined inflammation score that we found during the hospital admission but not at the six month follow-up are associated with the acute manic state.

Our study is limited by the relatively small sample size of the mania group and the fact that not all patients studied at the baseline visit were available for the six month follow-up visit. A more detailed study of individuals with mania at more frequent time points and with more immune markers and an investigation with larger numbers of bipolar disorder patients in well-characterized mood states studied longitudinally would provide additional information regarding the relationship between inflammatory markers and clinical course in individuals with bipolar disorder. The current study was meant to be exploratory and to encourage the combination of markers in future investigations. The elucidation of such markers might lead to new methods for predicting and monitoring the clinical course of individuals hospitalized with acute mania and for preventing relapse following hospital discharge. The establishment of inflammation in the etiopathogenesis of mania might also provide the rationale for studies of therapies directed at the modulation of this process. Such therapies might include newly developed anti-inflammatory compounds as well as interventions directed at altering the host microbiome which has been shown to be a major determinant of immune activation [22]. Such treatments may improve the clinical course and prevent illness exacerbation in individuals with this disorder.
